# Anthropometry in the immunotherapy of cutaneous and ocular melanomas

**Published:** 2020

**Authors:** Sorin Săftescu, Mihnea Munteanu, Dorel Popovici, Radu Dragomir, Maria Diana Dărăbuș, Alina Gabriela Negru, Șerban Mircea Negru

**Affiliations:** * OncoHelp Oncology Center, Timișoara, Romania; ** Department of Ophthalmology, “Victor Babeș” University of Medicine and Pharmacy, Timișoara, Romania; *** “Victor Babeș” University of Medicine and Pharmacy, Timișoara, Romania; **** Department of Cardiology, “Victor Babeș” University of Medicine and Pharmacy, Timișoara, Romania; ***** Department of Oncology, “Victor Babeș” University of Medicine and Pharmacy, Timișoara, Romania

**Keywords:** melanoma, nivolumab, anthropometry

## Abstract

The height of the adult individual is a balance of the expression of some genetic factors (especially the Y-M 170 haplotype of the Y chromosome) and the environment (nutrition and morbidity during childhood). Higher height is associated with a low risk of developing coronary heart disease, hypertension, gastroesophageal reflux, diaphragmatic hernia, but with a higher risk for atrial fibrillation, venous thromboembolism, intervertebral disc pathology, vasculitis and cancer. The research consisted of a retrospective observational study on patients who received immunotherapy (IT) with nivolumab for cutaneous and ocular melanoma neoplasms. We intended to highlight the associations between the duration of immunotherapy and sex profiles, age, anthropometric data (height, weight). Even though the number of available cases was relatively small (42), an inverse association between the body mass index of the subjects and the duration of immunotherapy could be proved, a more expressed association in case of male patients.

## Introduction

The height of the adult individual is a balance of the expression of some genetic and environmental factors. The most important non-genetic factors that determine the height of the adult individual are nutrition and morbidity during childhood (factors related to socio-economic, educational conditions). A lower height correlates with a lower average educational level and a lower social position as an adult [**1**].

The IM170 Y chromosome haplotype correlates with a higher average (unusually high) height of male individuals in a given population. The determinations reveal the highest proportions for this haplogroup in the countries of northern and south-eastern Europe. The average height for men in Sarajevo is 184 cm [**2**].

Higher height is associated with a low risk of developing coronary heart disease, hypertension, gastroesophageal reflux, diaphragmatic hernia, but with a higher risk of atrial fibrillation, venous thromboembolism, intervertebral disc pathology, vasculitis, cancer (odds ratio for models epidemiological and genetic, respectively: ORepi = 1.09, 95% CI 1.08–1.11; ORgen = 1.06, 95% CI 1.04–1.08) [**3**].

The average height of European populations in the Paleolithic (<9,000 BC) was estimated at 177 cm for males and 166 cm for females (higher than today's European average), to gradually decrease to averages of 161 cm for men and 154 cm for women in the late Neolithic (5000-3000 BC) [**4**]. The change seems to coincide with the shift from hunter-gatherers to farmers.

Also, the average life expectancy seems to have decreased from 33 years in the Paleolithic to 26 years in the Bronze Age and the Iron Age (3000 BC - 650 BC). However, given the high infant and pediatric mortality, the average life expectancy at birth does not give a fair overview of the historical situation. It is considered that people who reached the age of 15 (only 60% of those born) had a life expectancy of another 39 years (a total of 54 years) [**5**].

The historical evolution of the average height of European populations reveals an increase of 11 cm for males, born in 1870 and 1980, respectively, correlated with the improvement of the health status of the population expressed by the value of infant mortality [**6**].

However, after reaching maximum heights, around 2000 (the generation born in 1982), the average heights at 18 years old for both women and men suffered stagnation and even a modest decrease, which is true even for Romania and the country with the highest average height of the population - the Netherlands [**7**].

Until the introduction of immunotherapy, melanoma was considered a refractory disease marked by the lack of effective options in the treatment of metastatic disease. It was only after 2010, with the introduction of immunotherapy (anti-PD-1, anti-CTLA-4 antibodies), that a breach in the status of an unapproachable disease of metastatic melanoma was achieved. Immunotherapy response rates in metastatic melanoma can exceed 40%, with a remarkable percentage of long-lasting responses and good tolerability [**8**].

The combination of Nivolumab + Ipilimumab (anti-PD-1 + anti-CTLA-4 antibodies) manages to provide survival rates of over 50% at 5 years for cases of metastatic melanoma [**9**].

The rarity of cases of ocular melanoma makes it more difficult to assess the benefit of immunotherapy in this situation. Published data for cases of metastatic uveal melanoma reveal response rates of the order of 7% with disease control rates of 43%, and progression-free survival between 4 and 105 weeks (median 10 weeks). All these data describe uveal melanoma as relatively refractory to nivolumab immunotherapy [**10**].

Another study for patients with uveal melanoma using the combination of nivolumab and ipilimumab obtained response rates of 17% with disease control rates of 70%, with progression-free survival of 26 weeks, median survival of 83 weeks. However, the presence of immunotherapy-specific side effects was noted in 83% of the patients, with 40% of cases with grade 3 or 4, with 10% of the cases having treatment stopped due to side effects [**11**].

## Materials and methods

The research consisted of a retrospective observational study in patients who received immunotherapy (IT) with nivolumab for lung cancer, renal cancer and melanoma, seeking to highlight the associations between the duration of immunotherapy and sex, age, anthropometric data (height, weight).

Out of the 2241 hospitalizations (especially day-care) for the administration of immunotherapy belonging to 220 distinct persons between 28.06.2017 and 20.03.2020, attention was retained to the 42 cases of treated metastatic melanoma (including 2 cases of ocular melanoma). The administration of immunotherapy for melanoma cases started on 28.06.2017 and consisted of 527 administrations.

Because verification of the distribution of immunotherapy durations did not indicate a Gaussian type, the Mann-Whitney U Test (MWUT) was applied for sets of individual variables. The verification of the correlations between the duration of IT and various parameters was verified with Cox Proportional Hazards Survival Regression (CPHSR) which takes into account both cases still in treatment and those out of treatment.

## Results

Among 220 immunotherapeutic cases, the most extended recorded administration of immunotherapy is 783 days (patient with melanoma, treatment still in progress) and 35 cases benefited from only a single IT administration, another 21 cases being in treatment for less than 30 days.

**Fig. 1:**
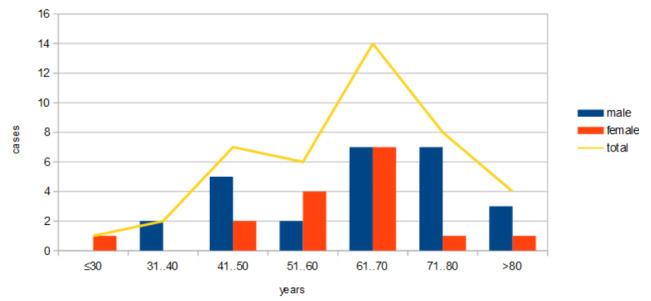
Distribution of melanoma cases by age and sex

Regarding the cases of cutaneous and ocular melanoma (a total of 42 cases, divided into 26 males and 16 females), the average duration of IT administration was 209 days (**Fig. 1**). Eighteen of the 42 cases of melanoma are still undergoing treatment. The mean age of the group of 42 patients with melanoma was 62 years for males and 58 years for females.

The distribution by age groups of melanoma cases indicated extreme values of 18 and 85 years, respectively, with the peak incidence located in the area of decades 7 and 8 (**Table 1**).

**Table 1:** Distribution of melanoma cases by age and sex

Age (years)	Male	Female	Total
≤30	0	1	1
31..40	2	0	2
41..50	5	2	7
51..60	2	4	6
61..70	7	7	14
71..80	7	1	8
>80	3	1	4

The distribution of cases according to the body mass index at the initiation of treatment reveals the dominance of patients with overweight and obesity, which had a much longer average duration of immunotherapy (**Table 2**).

**Table 2:** Melanoma cases and IT duration distribution by BMI

BMI (kg/ m2)	Cases	Average IT duration
20..24	6	5,83
25..29	17	5,4
30..34	7	15,62

Treated ocular melanomas are represented by two female cases, both 62 years old at initiation, treated with 6 and 4 IT administrations respectively, both off treatment at present due to progression.

**Fig. 2:**
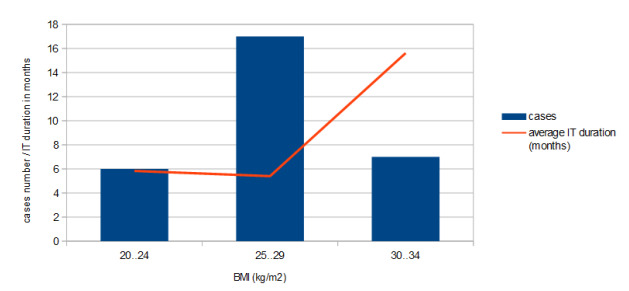
Melanoma cases and IT duration distribution by BMI

Of the 220 cases (lung + renal + melanoma), there were available data on the initiation weight for 174 cases, indicating an average weight of 74.15 kg (77.03 kg for males and 67.4 kg for females) (**Fig. 2**). 

**Fig. 3:**
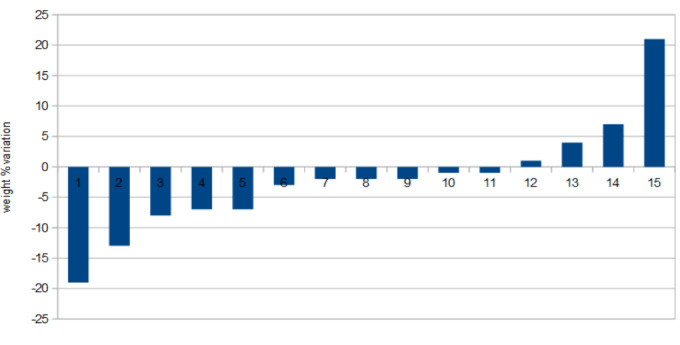
Relative weight variations during IT - cases of melanoma

For 45 cases, weight variation was recorded during treatment. The highest weight gain under treatment was + 57% (a case of kidney cancer), and the largest decrease was -22%, with an average of -1.15%. Two-thirds of cases suffered weight loss and one-third gained weight (among weight-weighted cases) (**Fig. 3**).

Among the cases of melanoma, we found 15 cases for whom the weight variation was documented and their evolution revealed a quarter of the cases with weight gain and approximately three quarters with weight loss.

The average duration of immunotherapy in cases whose weight has increased during treatment was 291 days, while for patients who lost weight, this average duration was 244 days. MWUT did not qualify the observed difference as statistically significant.

Cox Proportional Hazards Survival Regression was applied to different groups of cases, researching sex, age, height, initiation weight and body mass index as risk factors for shorter IT. Determinants for IT duration identified p values < 0.05 among cases with data available for height and weight. Interpretation: negative coefficients: IT duration increases with the determinant (favorable predictive factors); positive coefficients: IT duration decreases as the determinant increases (**Table 3**).

**Table 3:** Coefficients of variation for IT duration and statistical significance by different groups

Patient group	Parameter	Cases number	Coefficient	Statistical significance	Confidence interval	Risk Ratio	Confidence interval
Melanoma, available weight and height data cases at the initiation	BMI at initiation	30	-0.14	P=0.036	-0.26 to -0.09	0.87	0.76 to 0.99
Melanoma, available weight and height data cases at initiation, male cases	BMI at initiation	18	-0.28	P=0.029	-0.52 to -0.02	0.75	0.59 to 0.97

## Discussions

Fixed-dose administration of nivolumab in the immunotherapy of metastatic melanomas (and other cancers) would be expected to be more beneficial for anthropometrically less prominent individuals. Although a higher BMI does not automatically mean a higher clearance of the active substance, statistically we demonstrated an advantage over 10% per 1 unit increase of BMI for a longer duration of immunotherapy and implicitly for better survival. It should be noted that after the progression on treatment with immunotherapy it might be possible to continue with BRAF tyrosine kinase inhibitors (only for cases with documented BRAF mutation and if not already tried) and possibly chemotherapy (with uncertain effects on survival). A beneficial and more pronounced association of increased BMI throughout immunotherapy for male patients has been noted.

Without being able to reach statistical significance, other favorable predictors could be younger age, male gender and smaller height of the subjects but the p-value for these is between 0.19-0.25.

It seems that the dosage of immunotherapy still hides some mysteries.

## Conclusion

In the case of cutaneous and ocular melanomas, **the subject's body mass index is positively correlated with the duration of IT, with statistical significance (p <0.05)**.

Prolonged surveillance or a pool of cases of immunotherapeutic melanomas could statistically prove the value of other anthropometric determinants in terms of their correlation with the duration of immunotherapy.
